# Temporal visual processing deficits in post concussion syndrome

**DOI:** 10.1038/s41598-025-24029-0

**Published:** 2025-10-15

**Authors:** Davide Frattini, Mariagrazia Benassi, Tobias Wibble, Mattias Nilsson, Roberto Bolzani, Tony Pansell

**Affiliations:** 1 Department of Clinical Neuroscience, Karolinska Institutet, Eugeniavägen 12, Solna, 171 64 Sweden; 2 Department of Psychology, University of Bologna, Bologna, Italy

**Keywords:** Medical research, Neurology, Neuroscience, Psychology, Psychology

## Abstract

Post-concussive (PCS) motion hypersensitivity represents a common sequela of mild traumatic brain injury. This study investigated whether PCS alters visual temporal resolution thresholds in psychophysical measures that sustain motion detection. Fifteen PCS patients and fifteen age-matched controls underwent critical flicker fusion (CFF) threshold assessments across visual-field eccentricities. A Generalized linear mixed model tested group differences in CFF thresholds, treating eccentricity as a repeated factor and including CFF variability as a covariate. Pupil measurements and catch trials controlled for fatigue and alertness. Nonparametric correlations assessed relationships among time from injury, symptom severity, and CFF measures. Results showed CFF variability heightening CFF thresholds in the PCS group to a significantly larger extent compared to controls. Absence of significant CFF variability differences between groups, and modulation by eccentricity, suggests perceptual noise more strongly influences the overall visual temporal sensitivity in PCS. Days since injury negatively correlated with variability, indicating compensatory stabilization of temporal sensitivity over time. Symptom severity did not correlate with CFF measures. In conclusion, PCS motion hypersensitivity may reflect disturbances in visual temporal processing parameters, potentially involving altered internal neural noise. Although some recalibration occurs post-injury, persistent abnormalities underscore the need for further research into early, clinical interventions targeting perceptual noise.

**Supplementary Information:**

The online version contains supplementary material available at 10.1038/s41598-025-24029-0.

## Introduction

Visual motion hypersensitivity is a common sequela of mild head trauma, with up to 90% of affected individuals reporting such symptoms^1^. The acute underlying mechanism involves axolemmal stretching and electrochemical imbalances^2–4^, predominantly affecting heavily myelinated pathways spanning from subcortical to frontal and occipital-parietal regions^5,6^. These diffuse disruptions establish the basis for a complex symptom profile, ranging from cognitive impairments to multisensory dysfunction^7,8^. Although motion hypersensitivity frequently co-occurs with functional vestibular disturbances, structural alterations of the vestibular apparatus are not consistently observed. Instead, symptom manifestations appear more closely linked to hyperreactivity within cortical visual processing networks^9^ and impaired visual modulation of vestibular and oculomotor control centers^10^.

Historically, multisensory visual-vestibular integration in concussed and motion-hypersensitive individuals has been inferred through postural stability assessments^11^ and oculomotor proxies^10,12,13^. While these approaches are ecologically valid and reflect perceptual impairments, they are limited in their capacity to pinpoint the specific dysfunctional nodes within a distributed, multistage sensory processing network. Consequently, such methods provide a broad assessment but fall short of disentangling the discrete contributions of visual perception, multisensory integration, spatial orientation, and motor output processes.

In the present study, we examine one of the earliest stages of visual motion processing in individuals with post-concussion motion hypersensitivity. Specifically, we investigate thresholds for a key psychophysical metric, specifically the visual temporal resolution thresholds, that supports motion detection^14^. Visual temporal resolution, often termed “speed of vision”, is primarily constrained by the maximal spiking frequency of retinal ganglion cells, which defines the upper temporal boundary for stimulus integration in the visual pathway. Temporal integration of successive action potentials from these ganglion cells constitutes the neural basis of low-level motion perception. The visual system detects motion vectors and extrapolates object trajectories across brief temporal gaps by summing temporally adjacent spikes. Consequently, the intrinsic spiking dynamics of retinal ganglion cells establish a fundamental limit on the temporal fidelity with which visual events can be encoded and processed^14^. Visual temporal resolution limits can be tested via Critical Flicker Frequency (CFF), which is the high-frequency limit above which the response to a periodically modulated light cannot at any modulation amplitude be distinguished from the response to a steady field of the same mean luminance. While the retina’s temporal limits are defined by the electrophysiological responses of ganglion cells, human CFF testing has demonstrated that perceptual learning can elevate these thresholds, indicating neural integration and plasticity at post-retinal stages^15^. Post-retinal modulation is critically observed in the speed of visual processing, where the known electrophysiological advantage of peripheral over central regions becomes more pronounced when measuring visual processing speed through behavioral tasks^16^. Although previous results confirmed that temporal visual motion integration could explain visual motion hypersensitivity^17^ in post-concussion patients, little is known about the mechanism guiding these anomalies. Is it a matter of the spatial position of the stimuli, enhancing central or peripheral retinal response? Is the shift related to intermitted control, related to threshold variability or attention? We aimed to investigate whether anomalous shifts in visual temporal thresholds are specific for peripheral regions of the visual field and if it is related to threshold variability. Building on evidence of increased activity in visual cortices^9^ and heightened vestibular-oculomotor responsiveness during optokinetic stimulation^10,12,13^, we hypothesize that shifts in visual temporal thresholds may be present, and possibly elevated, in peripheral regions of the visual field. In these areas, where multisensory integration plays a significant role in postural control and the sense of vection, such alterations may contribute to the atypical motion integration driving non-vestibular vertigo observed in this population. Moreover, we examine the link between these anomalies and symptoms severity.

## Methods

### Participants

Fifteen patients with persistent post-concussion symptoms (PCS) (10 female, mean age = 48.47) were recruited from local neurorehabilitation and optometry clinics, along with fifteen age-matched healthy controls (6 female, mean age = 38.53) recruited through the Karolinska Institutet research participation portal. Inclusion criteria for the PCS group were: (I) a clinical diagnosis of concussion ≥ 3 months prior by a licensed neurologist, (II) negative neuroimaging results, (III) continued adherence to PCS criteria according to the International Classification of Diseases, 10th Revision (ICD-10), with persistent visual motion-induced discomfort and dizziness at the time of testing. The mean time since injury was 44.1 ± 44.4 months. All patients underwent prior clinical assessment and met established PCS classification guidelines. All participants provided written informed consent, and the study was conducted in accordance with the Declaration of Helsinki. Participant inclusion, data collection, handling of personal data and the experimental protocol setup have been approved by the Swedish Ethics Review Authority (ethics review board approval: 2023-06696-02).

Before inclusion, each participant completed a comprehensive clinical testing battery. Exclusion criteria were: (I) impaired ocular motor control or gaze function as determined by clinical ocular motility examination, (II) any documented vestibular, somatosensory deficits, (III) recent initiation of medications affecting the central nervous system (< 3 months), and (IV) any diagnosed neurological or sensory condition other than PCS. Eye examinations ensured normal ocular motility and stereoscopic vision (TNO test ≤ 60 arcsec). Peripheral vestibular integrity was confirmed via head impulse tests in all semicircular canal planes. Central vestibular ocular motor function was assessed by verifying the absence of skew deviation. Somatosensory contributions to balance were evaluated with an enforced Romberg’s test.

### Symptom assessment

A neuropsychologist conducted non-directive interviews to confirm PCS status according to ICD-10 criteria, relying on patient clinical history and subjective symptomatology. All PCS patients had been undergoing visual rehabilitation therapy initiated within the previous year, and none reported concurrent interventions. Interviews also provided educational and professional background details to match with controls. The Situational Vertigo Questionnaire (SVQ) was administered to quantify symptom severity^18^ (see supplementary materials). The chronicity of PCS was quantified as the number of days since injury (DFI), calculated as the time elapsed between the concussion date recorded in the national medical journal and the testing date. Two patients were excluded: one due to pre-existing abnormal visual development and one because neurological examination data were unavailable in national medical records.

### Flicker stimulation instrument

Flicker stimulation was generated by a LED (Bivar Orca R R20WHT-F-0160) controlled by a multifunction I/O device (NI USB-6001). Both the LED and the control device were housed in a 3D-printed enclosure. An adjustable aperture regulated the exit diameter of the luminous flux, and an opal glass filter ensured uniform light distribution.

The LED luminance was sinusoidally modulated around a baseline luminance (L_0_ = 50 cd/m^2^) with an amplitude modulation (m = 1) for 100% modulation depth, producing luminance peaks of 100 cd/m^2^ and troughs of 0 cd/m^2^. Flicker frequency (f) was measured in Hertz (Hz), with each stimulation lasting 1 s. The analog signal was defined as:

L_(t)_ = L_0_ × (1 + m sin (2π ft)).

Ambient retinal illuminance was maintained at 30 lx (≈ 30 trolands, assuming a 2 mm pupil), whereas the background luminance was 2 cd/m^2^. Uniformity of the luminous flux and accuracy of the flicker waveform were verified with a photometer (Hagner Universal Photometer S4) connected to an oscilloscope (Tektronix TBS1052C).

### Critical flicker frequency (CFF) assessment

Circular flicker stimuli were presented monocularly at eccentricities of 5°, 10°, 20°, and 40° from fixation, on both nasal and temporal sides of the visual field resulting in total eight stimulation positions, with the participant head positioned on a chinrest 60 cm from the flicker source. Stimulus size was M-scaled according to cortical magnification factors^19^ to maintain a constant cortical projection area across eccentricities, resulting in visual angle diameters (VADs) of 0.46°, 0.74°, 1.31°, and 2.55° at 5°, 10°, 20°, and 40°, respectively.

A 120 Hz flickering fixation target (0.19° VAD) remained above threshold to facilitate stable fixation and used as reference to determine flickering behavior of the target stimulus. Prior to each test, a 1-minute adaptation period at the given eccentricity was implemented, during which the LED flickered at 120 Hz with mean luminance L_0_ on both target and fixation fields, ensuring uniform retinal adaptation. Participants were tested with normal or corrected to normal visual acuity to ensure equal retinal stimulation during the testing.

**Fig. 1:**
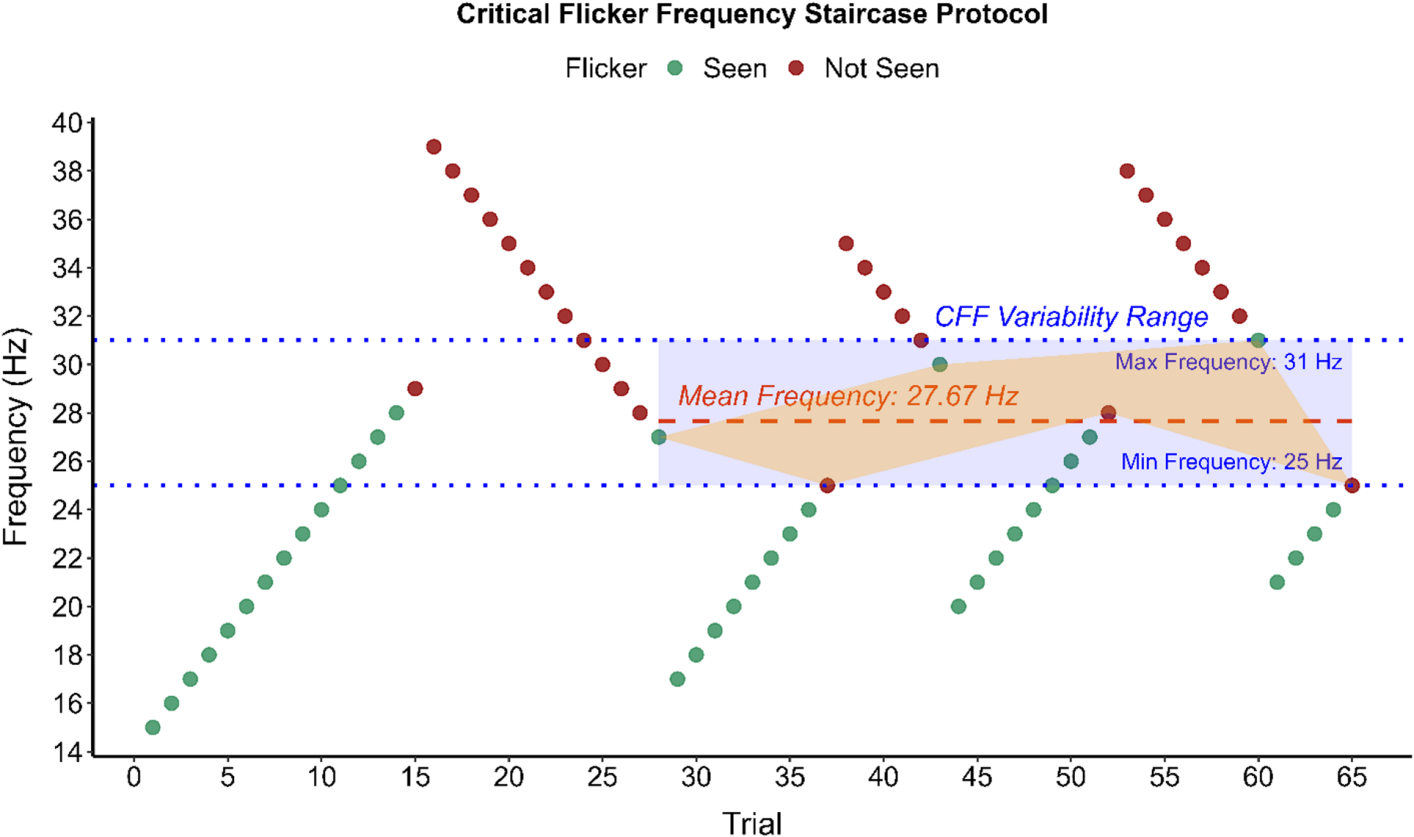
The staircase critical flicker frequency assessment protocol. A scatterplot illustrating the Critical Flicker Frequency threshold assessment of a control subject at 10° from fixation. As illustrated, the first leg of the staircase is discarded and the CFF threshold is represented by the mean of the six subsequent thresholds levels, one in each staircase leg (orange). The CFF variability score (blue) is retrieved from the minimum and maximum frequency thresholds within the six staircase that are retrieved to calculate the man CFF threshold.

CFF thresholds were determined using a staircase procedure. Starting from an upward staircase with perceptible flicker frequency for each eccentricity, based on pilot data, frequency was increased by a step of 1 Hz per trial until participants reported the flicker becoming imperceptible. Every upward staircase was followed by a downward staircase, and vice versa, in which the initial flicker frequency was upregulated by an additional 10 Hz from the previous upward staircase threshold before reversing and decreasing until the flicker again became perceptible. The same procedure, with reversed 10 Hz frequency downregulation, was applied for upward staircase following a downward staircase leg (see Fig. 1). Attention was monitored via catch trials (120 Hz flicker) interspersed every 6–10 trials; failure to detect absence of flicker in these trials invalidated the current staircase leg, prompting an additional measurement. Correct gazing at the fixation target was monitored with eye tracking at 1000 Hz (SR Eyelink 1000 Plus). Flicker signal generation, eye-tracking synchronization, response logging, and staircase progression were controlled with a MATLAB script (MATLAB R2024b, The MathWorks, Inc.).

### Measurements

A total of seven staircase legs were administered, with the initial leg excluded from analysis. CFF thresholds were calculated as the average of the six threshold values, three from the ascending leg and three from the descending leg of the staircase. Pupil diameter was recorded concurrently, and its average was computed across threshold trials. A CFF variability score, defined as the range between the maximum and minimum threshold values within the six staircase legs, was derived according to established guidelines^20^ (see Fig. 1 and see Table 1).

**Table 1:** Descriptive data of the CFF threshold and CFF variability range, as mean and 95% CI, for the PCS group and control group within each level of the eccentricity of flicker stimulation.

Eccentricity (º)	Control		PCS	
	Threshold (Hz)	Variability (Hz)	Threshold (Hz)	Variability (Hz)
5	26.54 (24.30–28.77)	7.37 (6.01–8.73)	27.27 (25.03–29.50)	7.90 (6.54–9.26)
10	31.83 (29.23–34.44)	9.17 (7.73–10.61)	32.77 (30.16–35.37)	10.17 (8.73–11.61)
20	40.38 (37.29–43.47)	8.77 (7.32–10.22)	42.68 (39.59–45.77)	9.87 (8.42–11.32)
40	48.16 (44.47–51.85)	9.03 (7.18–10.16)	54.24 (50.43–58.06)	12.07 (10.88–13.99)

### Statistical analysis

All analyses were conducted using SPSS Statistics 28 (IBM Corp., Armonk, NY, USA). A two-tailed significance alpha level of 0.05 was set for all the statistical tests executed in the analysis. Power Analysis was tested on the final CFF threshold model with an R script (lme and simr packages). The power analysis model was tested with subject-level covariates to replicate the Generalized Linear Mixed Model (GLMM). The designed parameters were two groups (15 participants each), four repeated-measures levels (eccentricity factor), Rho equals to 0.77 as observed in the compound-symmetry correlation among measures, effect size (beta of the slope) equals to 0.20, with a total number of simulations set to five hundreds. The model achieves 100.0% power (95% CI: 99.26–100.0).

### Preliminary analysis

**Fig. 2:**
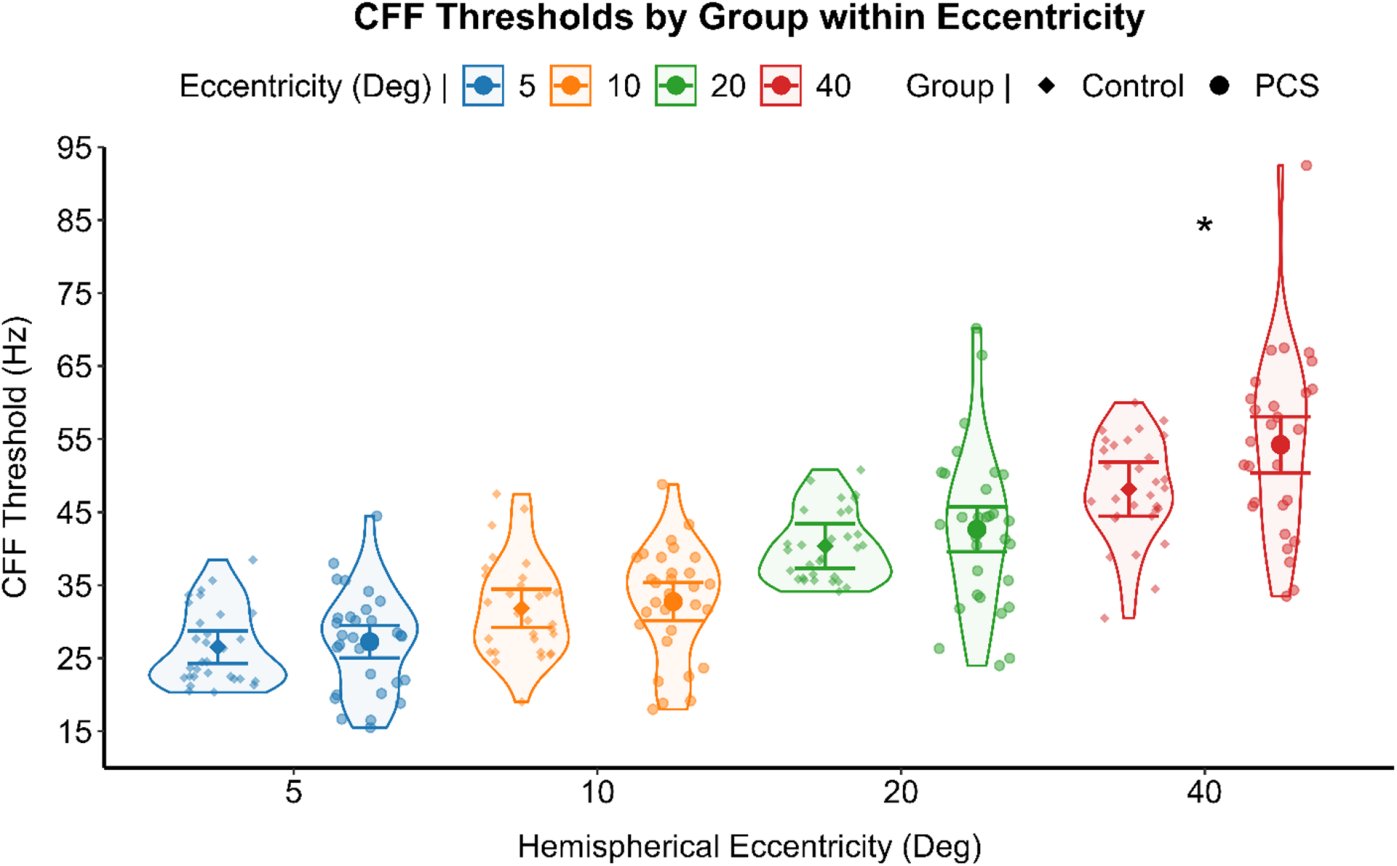
Preliminary analysis results regarding CFF threshold between group and eccentricities. A violin scatter plot representing the CFF thresholds of each subject (scatterplot dots). The results of the Anova for each level of the eccentricity of stimulation is represented by the interval plot, with the means (dot) with 95% CI (error bars). * *p* = .025.

**Fig. 3:**
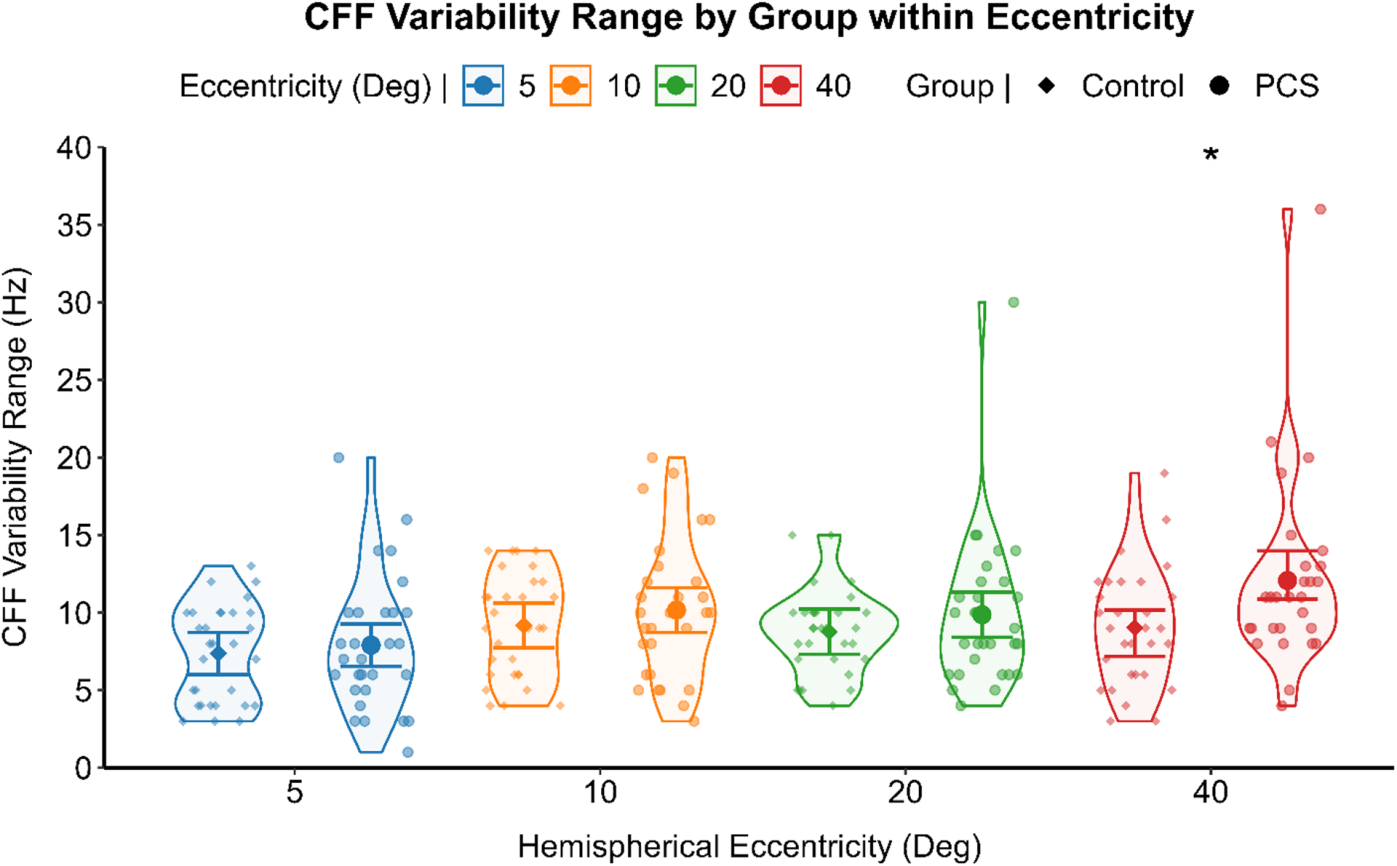
Preliminary analysis results regarding CFF variability between group and eccentricities. A violin scatter plot representing the CFF variability range of each subject (scatterplot dots). The results of the Anova for each level of the eccentricity of stimulation is represented by the interval plot, with the means (dot) with 95% CI (error bars). *p = .026.

**Fig. 4:**
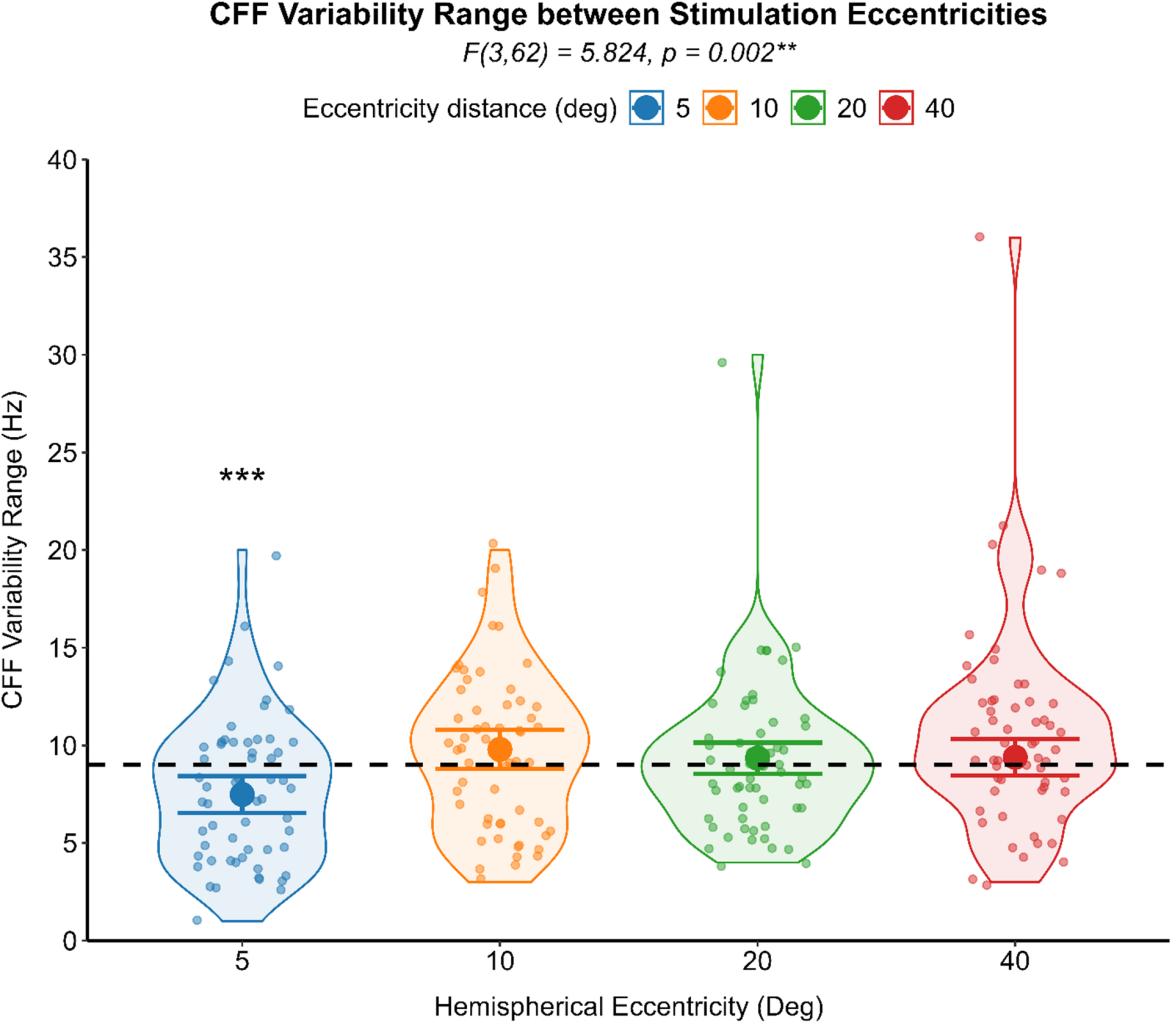
Results of the analysis regarding the effect of eccentricity over the CFF variability. A violin scatter plot representing the CFF variability range of each subject (scatterplot dots) is superimposed onto the interval plot representing the estimated marginal mean (dot) with 95% CI (error bars) as the difference from the overall mean (dash line) for each level of eccentricity of flicker stimulation. **p < .01, *** *p* < .001.

The critical flicker fusion (CFF) thresholds and the CFF variability score relationship, assessed through non-parametric correlation for each group, indicated a significant positive correlation between the two psychometric measures, in the healthy (Spearman’s ρ (120) = 0.214, *p* = .019), and the PCS group (Spearman’s ρ (118) = 0.381, *p* < .001). Differences in CFF thresholds and in CFF variability scores were analyzed by separated GLM for each level of the eccentricity of stimulation. PCS individuals reported significant higher CFF thresholds (F (1,56) = 5.270, *p* = .025) (see Fig. 2) and higher CFF variability score (F (1,56) = 5.225, *p* = .026) (see Fig. 3) at the most peripheral eccentricity of stimulation, compared to healthy individuals. A generalized linear mixed model (GLMM) with an identity link function was employed to compare CFF variability (dependent variable) between PCS and healthy controls using the group as between-subject factor. Participants were included as a random effect, and visual field and eccentricity were modeled as repeated measures, with pupil size as a covariate. When controlling for pupil size and inter-individual variability with subjects being a random factor, CFF variability did not differ significantly between groups (F (1,25) = 1.480, *p* = .235) with healthy individuals (Mean = 8.39, 95% CI [2.08, 14.70]) and PCS individuals (Mean = 9.57, 95% CI [3.25, 15.89]) reporting comparable results. CFF variability, on the other hand, was significantly affected by the eccentricity of the stimulation (F (3,52) = 5.824, *p* = .002). Post hoc analysis deviation from the mean contrast with sequential Bonferroni correction for multiple comparison revealed a significant lower CFF variability only in the most central stimulation (Mean Difference = -1.517, SE = 0.36, 95% CI [-2.45, -0.57], *p* < .001) (Fig. 4).

### CFF threshold model

In line with the analysis reported over the CFF variability score, a generalized linear mixed model (GLMM) with identity link function was employed to compare critical flicker fusion (CFF) thresholds (dependent variable) and groups (patients with persistent post-concussion symptoms (PCS) and healthy controls) as between-subject factor. Participant was included as a random factor, and visual field and eccentricity were modeled as repeated measures. The group, eccentricity and the interaction between group by eccentricity were considered as fixed effects in the model. Mean pupil size and CFF variability range were considered as covariates and included in the model ad interacting with group factor. To assess relationships between symptoms and CFF measures within the PCS group, Spearman’s correlation analysis was used to assess the relationship between symptom severity (SVQ scores) and time since injury to CFF thresholds and CFF variability.

## Results

**Fig. 5:**
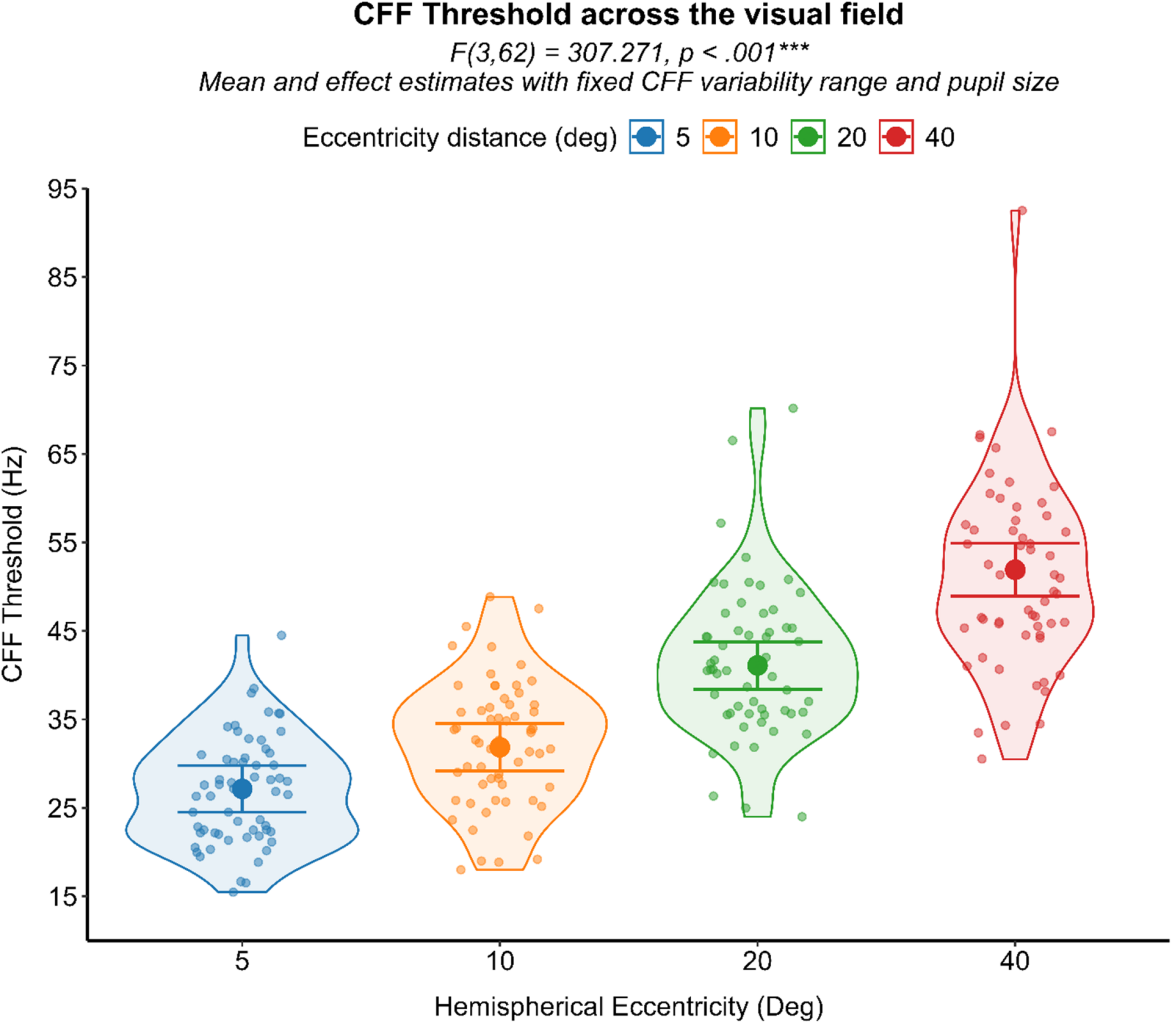
Results of the analysis regarding the effect of eccentricity over the CFF threshold. A violin scatter plot representing the CFF Threshold of each subject (scatterplot dots) is superimposed onto the interval plot representing the estimated marginal mean (dot) with 95% CI (error bars) from the GLMM analysis with fixed CFF variability and pupil. ***p < .001.

From the Generalized linear mixed model, eccentricity significantly influenced CFF thresholds (F (3,62) = 307.271, *p* < .001), indicating that peripheral visual field locations were associated with higher temporal resolution processing compared to more central regions. This finding, observed independently of group membership, aligns with established previous literature, supporting the validity of the current custom-made experimental apparatus and the faster visual processing at peripheral over central visual field^18^. (Fig. 5).

**Fig. 6:**
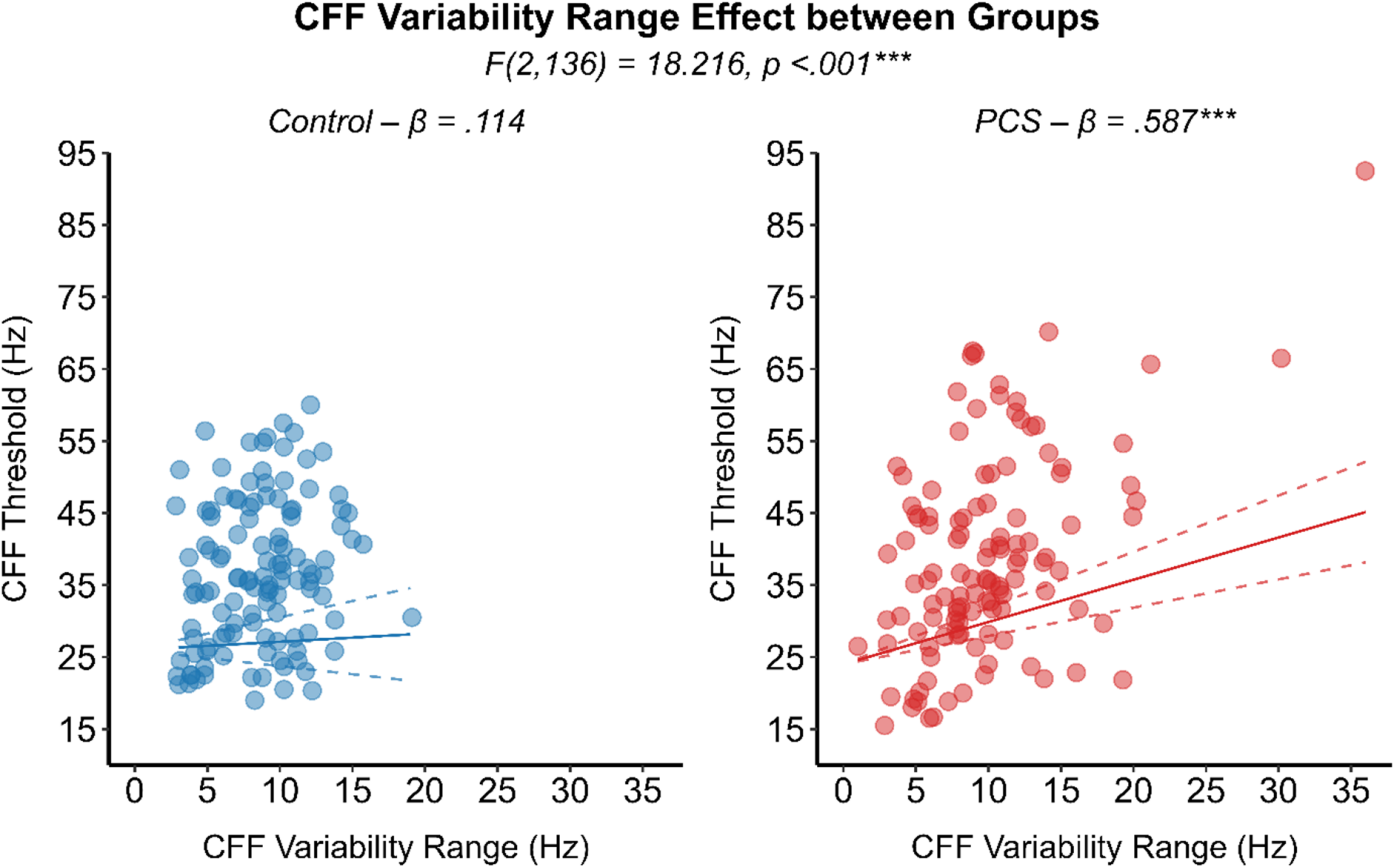
Results of the analysis regarding the interacting effect of the covariate CFF variability over the CFF threshold between groups. A scatterplot, with datapoint indicating each subject mean CFF from all six staircase legs for a given eccentricity stimulation. The significant interaction effect between group and CFF variability range, with fixed pupil, is represented by the regression line illustrating the significant modulating effect of CFF variability over the CFF threshold in the PCS group (red circle) compared to the control group (blue circle). ***p < .001.

**Fig. 7:**
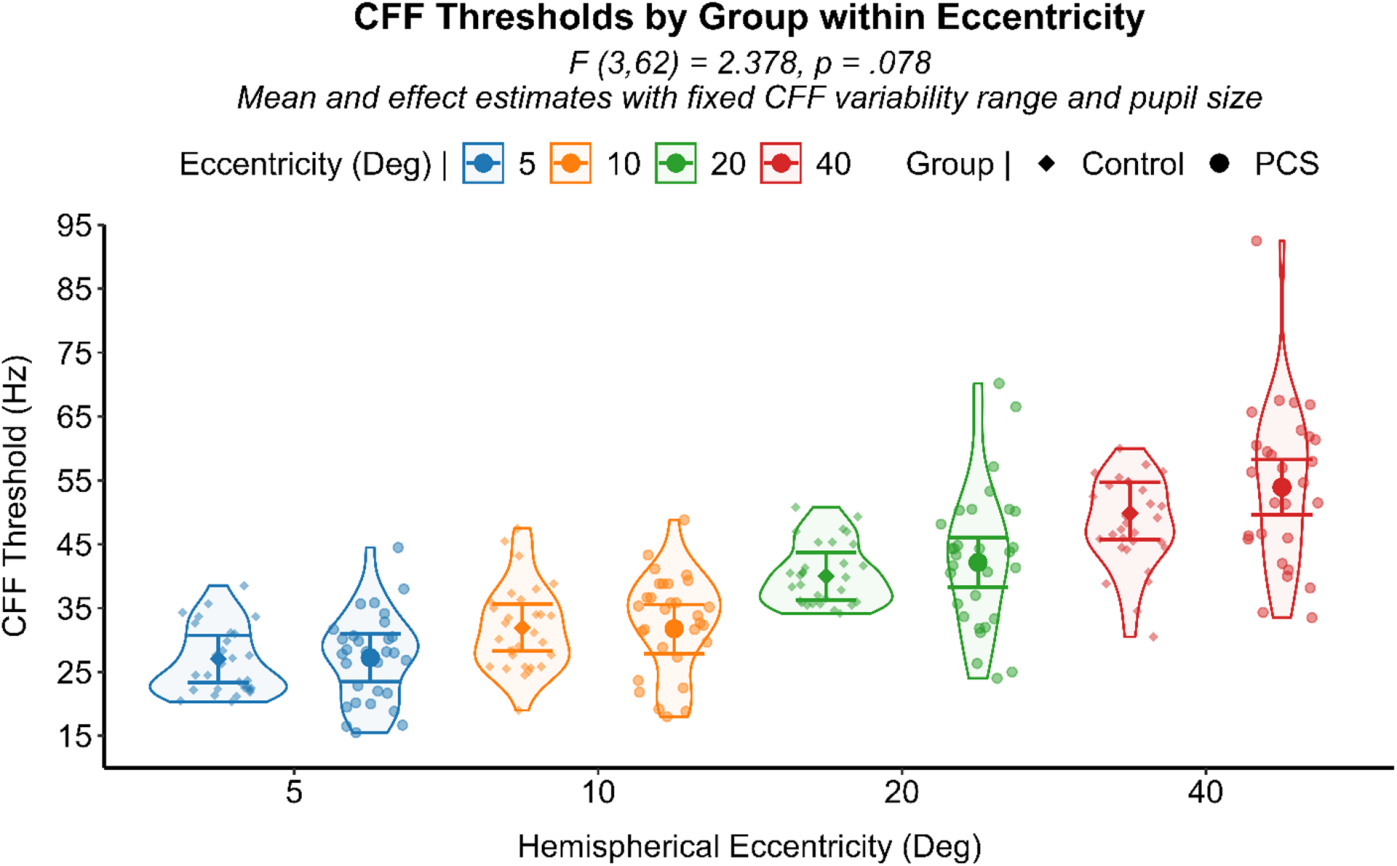
Results of the analysis regarding the interacting effect of group and eccentricity over the CFF threshold. A violin scatter plot representing the CFF Threshold of each subject (scatterplot dots) is superimposed onto the interval plot representing group differences within stimulation eccentricities with the estimated marginal mean (dot) and 95% CI (error bars) from the GLMM analysis with fixed CFF variability and pupil.

Moreover, a significant interaction effect between CFF variability and groups was noted (F (2,136) = 18.216, *p* < .001). Specifically, CFF variability heightened CFF thresholds in the PCS group to a significantly larger extend (β = 0.587, SE = 0.09, 95% CI [0.39, 0.78], *p* < .001) compared to healthy controls (β = 0.114, SE = 0.17, 95% CI [-0.22, 0.45], *p* = .505) (Fig. 6). Although CFF variability and CFF thresholds were positively correlated in both groups, its influence in heightening the CFF threshold was five times higher in the PCS group. Comparable CFF variability between groups, along with its significantly reduced magnitude in visual field regions with higher signal-to-noise ratios, suggests that perceptual noise may underlay the observed CFF variability which in turn significantly heightened the CFF thresholds in the PCS group. By evaluating all eccentricity levels together and adjusting for CFF variability and pupil size as covariates, PCS individuals showed a trend of increased CFF thresholds toward the periphery compared to the control group (F (3,62) = 2.378, *p* = .078) (see Fig. 7). Spearman’s rank-order correlation analysis revealed a significant negative relationship between days from injury (DFI) and CFF variability (ρ (118) = − 0.212, *p* = .021), indicating that individuals with longer post-injury intervals tended to exhibit lower variability in their CFF thresholds. On the other hand, subjective symptomatic questionnaire showed no relationship with any temporal resolution processing measurements.

## Discussion

This study sought to elucidate the role of low-level visual motion processing alterations in individuals with persistent post-concussion motion sensitivity. Our findings confirmed that visual temporal resolution thresholds (CFF) increase with retinal eccentricity, consistent with previous literature, thus supporting the reliability of our custom-made LED flicker stimulator^19^. This effect was present regardless of PCS status, reinforcing the notion that fundamental sensory and perceptual temporal processing properties remain intact and follow typical spatial patterns^16^.

Our analysis revealed that perceptual noise, in the shape of variability of CFF thresholds within the same stimulation block, strongly affects visual temporal resolution thresholds in the PCS population. Although perceptual noise is present to a similar degree in both populations and generally increases the temporal resolution thresholds in both populations, the magnitude of such increment was five times higher for the PCS population. These results suggest that patients who have sustained mild head trauma may be subjected to subtle disturbances in the earliest stages of visual motion processing. Specifically, the observation that higher visual temporal sensitivity thresholds are driven by perceptual noise hints at alterations in signal transmission and neuronal firing patterns. Functional and structural connectivity alterations within the visual system have already been described, supporting the notion of altered signal transmission^6,21^. Additionally, several studies have reported alterations in event-related potentials (ERPs) within the visual cortex, indicating lower signal transmission strength along with higher neuronal noise^22,23^. A potential explanatory framework for the visual temporal sensitivity shifts due to perceptual noise observed in the current study may lie in the concept of stochastic resonance and the role of internal neural noise. Previous computational models and neurophysiological studies suggest that low-level neural noise can modify neuronal firing thresholds, effectively shifting sensitivity ranges^24,25^. In a healthy neural system, moderate levels of internal noise can enhance signal detection under certain conditions, but in a concussed brain, dysregulated noise levels may destabilize normal threshold settings by flattening the sigmoid function that generally shape the neuronal firing probability and signal detection^24^. This perturbation could impair the tuning of early perceptual filters, increasing patients’ reactivity to visual motion and thereby making them more susceptible to motion-induced discomfort. As sensitivity weights are shifted, the input to multisensory integration processes becomes less coherent, further exacerbating perceptual instability.

The presence of heightened visual temporal sensitivity, driven by perceptual noise, suggests that the local visual network may be operating in a state of increased irregularity. In such a more chaotic setting, facilitated diffusion can enhance signal propagation across the network and related pathways^24^. Similar phenomena have been observed in prior investigations demonstrating that increased visual motion sensitivity can influence vestibular and oculomotor function^10^. Although overt structural damage to the vestibular apparatus and oculomotor control centers may be absent, their effective operation relies on coherent visual input. The ability to detect motion, as we tested through the temporal resolution processing limits, does not imply the ability of the subject to have accurate motion extrapolation. Despite previous literature supporting the positive relationship between visual processing speed and motion detection^15^, the heightened temporal resolution processing observed in the PCS is primarily driven by interval noise. The trend of increasing temporal resolution processing in peripheral areas where the signal to noise ratio is generally decreased exacerbates the effect of noise in modulating the sensory threshold, though such effect is already present independently of stimulation eccentricity in the PCS population. Whereas temporal resolution processing seems to play a theoretical advantage for the PCS population, motion detection and motion extrapolation in ecological setting requires the visual system to accurately encode the motion signal and vectors while dampening the influence of background noise. Such concept is particularly evident in other similar clinical populations with visual motion hypersensitivity, where the ability to extrapolate coherent motion direction from background noise is impacted compared to healthy controls^26^. As a result, the factors modulation sensitivity thresholds and motion extrapolation ability in PCS population hint to an overly noisy visual motion system.

The observed negative correlation between chronicity (days from injury) and CFF variability, though weak, implies that over time, partial neurological recalibration or compensatory processes may reduce this irregularity. Longer post-injury intervals could enable residual neural circuits to adjust, partially re-establishing more stable temporal resolution thresholds by decreasing internal noise. Nonetheless, the persistence of abnormal motion sensitivity in numerous patients indicates that these compensatory adaptations remain incomplete and warrant further investigation.

Several limitations must be acknowledged. The sample size, although reporting adequate power for the study, was relatively small, and our measurements focused primarily on behavioral and perceptual outcomes rather than direct neurophysiological recordings. Consequently, we cannot definitively pinpoint the precise neuronal mechanisms or brain regions responsible for the observed variability. Future studies employing advanced imaging modalities or neurophysiological assessments could provide a more detailed picture of these processes. Additionally, participants were undergoing various stages of rehabilitation, potentially influencing their perceptual performance and the plasticity of their sensory systems^27^. This notion is further supported by the observed decrease in threshold instability with increasing time since injury. To account for inter-individual variability, each participant was included as a random factor in the model, thereby enabling the assessment of effects at the individual level. While factors such as fatigue and alertness are common in PCS^28^, these influences were monitored and controlled using catch trials and concurrent pupil measurements. The comparable pupil sizes across groups suggest similar levels of fatigue and vigilance, and CFF outcomes were accordingly adjusted for pupil size by setting pupil metrics as covariates in the statistics model, ensuring that observed differences in temporal resolution were not driven by these confounding factors.

In conclusion, our findings support the notion that post-concussion motion hypersensitivity may stem from subtle alterations in low-level visual temporal processing. The data points toward an interplay between internal neural noise and temporal resolution thresholds, influencing how visual motion signals are detected and integrated. As these disruptions may persist over time, even if partially attenuated with increasing post-injury intervals, examining targeted interventions aimed at reducing perceptual noise may serve to improve patient care. Specifically, in addition to optometric rehabilitation^27^, which addresses binocular, oculomotor, and accommodative functions commonly impaired after concussion, incorporating motion-direction learning tasks, such as those proposed by Seitz et al.^15^, may enhance visual attention and sensory learning. In these protocols, attentional tasks performed in the presence of background motion have been shown to improve motion-coherence thresholds, directly indexing the ability to detect meaningful motion signals amid noise. Ultimately, these protocols increased critical flicker fusion (CFF) thresholds; although variability was not assessed, the findings support a link between flicker perception and motion detection. Such approaches may ultimately enhance rehabilitation strategies and improve clinical outcomes for individuals experiencing persistent motion sensitivity following mild head trauma.

## Supplementary Information

Below is the link to the electronic supplementary material.

Supplementary Material 1

## Data Availability

The datasets used and/or analyzed during the current study are available from the corresponding author on reasonable request.

## References

[1] Lumba-Brown, A., Niknam, K., Cornwell, J., Meyer, C. & Ghajar, J. Sex-Related differences in neurosensory alterations following blunt head injury. *Front. Neurol.* **11**, 1051. 10.3389/fneur.2020.01051 (2020). 33041988 10.3389/fneur.2020.01051 PMC7522405

[2] Katz, D. I., Cohen, S. I. & Alexander, M. P. Mild traumatic brain injury. *Handb. Clin. Neurol.* **127**, 131–156. 10.1016/B978-0-444-52892-6.00009-X (2015). 25702214 10.1016/B978-0-444-52892-6.00009-X

[3] Bigler, E. D. Neuropathology of mild traumatic brain injury: correlation to neurocognitive and neurobehavioral findings. In *Brain Neurotrauma: Molecular, Neuropsychological, and Rehabilitation Aspects* (ed Kobeissy, F. H.) (CRC Press/Taylor & Francis, 2015). 26269912

[4] Govind, V. et al. Whole-brain proton MR spectroscopic imaging of mild-to-moderate traumatic brain injury and correlation with neuropsychological deficits. *J. Neurotrauma*. **27**(3), 483–496. 10.1089/neu.2009.1159 (2010). 20201668 10.1089/neu.2009.1159 PMC2867627

[5] Bigler, E. D. & Maxwell, W. L. Neuropathology of mild traumatic brain injury: relationship to neuroimaging findings. *Brain Imaging Behav.* **6**(2), 108–136. 10.1007/s11682-011-9145-0 (2012). 22434552 10.1007/s11682-011-9145-0

[6] Van Ombergen, A. Altered functional brain connectivity in patients with visually induced Dizziness. *NeuroImage Clin.* **14**, 538–545. 10.1016/j.nicl.2017.02.020 (2017). 28331800 10.1016/j.nicl.2017.02.020 PMC5345975

[7] Wibble, T. & Pansell, T. Clinical characteristics of visual motion hypersensitivity: a systematic review. *Exp. Brain Res.* **241**(7), 1707–1719. 10.1007/s00221-023-06652-3 (2023). 37341755 10.1007/s00221-023-06652-3 PMC10349011

[8] Dwyer, B. & Katz, D. I. Postconcussion syndrome. *Handb. Clin. Neurol.* **158**, 163–178 (2018). 10.1016/B978-0-444-63954-7.00017-3 10.1016/B978-0-444-63954-7.00017-3 30482344

[9] Astafiev, S. V., Zinn, K. L., Shulman, G. L. & Corbetta, M. Exploring the physiological correlates of chronic mild traumatic brain injury symptoms. *NeuroImage Clin.* **11**, 10–19. 10.1016/j.nicl.2016.01.004 (2016). 26909324 10.1016/j.nicl.2016.01.004 PMC4732189

[10] Frattini, D., Rosén, N. & Wibble, T. A proposed mechanism for visual vertigo: Post-Concussion patients have higher gain from visual input into subcortical gaze stabilization. *Investig. Ophthalmol. Vis. Sci.* **65**(4), 26. 10.1167/iovs.65.4.26 (2024). 10.1167/iovs.65.4.26 PMC11018265 38607620

[11] Bronstein, A. M. Vision and vertigo: some visual aspects of vestibular disorders. *J. Neurol.* **251**(4), 381–387. 10.1007/s00415-004-0410-7 (2004). 15083281 10.1007/s00415-004-0410-7

[12] Bertolini, G., Romano, F., Straumann, D., Keller, K. & Palla, A. Feddermann-Demont, N. Measuring optokinetic after-nystagmus: potential for detecting patients with signs of visual dependence following concussion. *J. Neurol.* **268**(5), 1747–1761. 10.1007/s00415-020-10359-8 (2021). 33367947 10.1007/s00415-020-10359-8 PMC8068696

[13] Wibble, T., Frattini, D., Benassi, M., Bolzani, R. & Pansell, T. Concussed patients with visually induced Dizziness exhibit increased ocular torsion and vertical vergence during optokinetic gaze-stabilization. *Sci. Rep.* **13**(1), 3690. 10.1038/s41598-023-30668-y (2023). 36879031 10.1038/s41598-023-30668-y PMC9988826

[14] Donner, K. Temporal vision: measures, mechanisms and meaning. *J. Exp. Biol.* **224**(15), jeb222679. 10.1242/jeb.222679 (2021). 34328511 10.1242/jeb.222679 PMC8353166

[15] Seitz, A. R., Nanez, J. E., Sr, Holloway, S. R. & Watanabe, T. Perceptual learning of motion leads to faster flicker perception. *PloS One*. **1**(1), e28. 10.1371/journal.pone.0000028 (2006). 17183655 10.1371/journal.pone.0000028 PMC1762365

[16] Carrasco, M., McElree, B., Denisova, K. & Giordano, A. M. Speed of visual processing increases with eccentricity. *Nat. Neurosci.* **6**(7), 699–700. 10.1038/nn1079 (2003). 12819786 10.1038/nn1079 PMC3077107

[17] Chang, T. T., Ciuffreda, K. J. & Kapoor, N. Critical flicker frequency and related symptoms in mild traumatic brain injury. *Brain Inj.* **21**(10), 1055–1062. 10.1080/02699050701591437 (2007). 17891568 10.1080/02699050701591437

[18] Guerraz, M. et al. Visual vertigo: symptom assessment, Spatial orientation and postural control. *Brain* **124**(8), 1646–1656. 10.1093/brain/124.8.1646 (2001). 11459755 10.1093/brain/124.8.1646

[19] Rovamo, J. & Raninen, A. Critical flicker frequency and M-scaling of stimulus size and retinal illuminance. *Vision. Res.* **24**(10), 1127–1131. 10.1016/0042-6989(84)90166-4 (1984). 6523734 10.1016/0042-6989(84)90166-4

[20] Ákos, K. & Ákos, M. *The Critical Flicker Frequency Series Effect* (Akadémiai Kiadó, 1966).

[21] Gilmore, C. S. et al. Deficits in visual system functional connectivity after Blast-Related mild TBI are associated with injury severity and executive dysfunction. *Brain Behav.* **6**(5), e00454. 10.1002/brb3.454 (2016). 27257516 10.1002/brb3.454 PMC4873652

[22] Yadav, N. K. & Ciuffreda, K. J. Objective assessment of visual attention in mild traumatic brain injury (mTBI) using visual-evoked potentials (VEP). *Brain Inj.* **29**(3), 352–365. 10.3109/02699052.2014.979229 (2015). 25415539 10.3109/02699052.2014.979229

[23] Fong, D. H. C. et al. Steady-State Visual-Evoked potentials as a biomarker for concussion: A pilot study. *Front. NeuroSci.* **14**, 171. 10.3389/fnins.2020.00171 (2020). 32210749 10.3389/fnins.2020.00171 PMC7076115

[24] Destexhe, A. & Contreras, D. Neuronal computations with stochastic network States. *Science* **314**(5796), 85–90. 10.1126/science.1127241 (2006). 17023650 10.1126/science.1127241

[25] van der Groen, O. & Wenderoth, N. Transcranial random noise stimulation of visual cortex: stochastic resonance enhances central mechanisms of perception. *J. Neurosci.* **36**(19), 5289–5298. 10.1523/JNEUROSCI.4519-15.2016 (2016). 27170126 10.1523/JNEUROSCI.4519-15.2016 PMC6601807

[26] Storm, R. et al. Visual and vestibular motion perception in persistent postural-perceptual Dizziness (PPPD). *J. Neurol.* **271**(6), 3227–3238. 10.1007/s00415-024-12255-x (2024). 38441610 10.1007/s00415-024-12255-x PMC11136745

[27] Freed, S. & Hellerstein, L. F. Visual electrodiagnostic findings in mild traumatic brain injury. *Brain Inj.* **11**(1), 25–36. 10.1080/026990597123782 (1997). 9012549 10.1080/026990597123782

[28] McCrory, P. et al. Consensus statement on concussion in sport-the 5th international conference on concussion in sport held in Berlin, October 2016. *Br. J. Sports Med.* **51**(11), 838–847. 10.1136/bjsports-2017-097699 (2017). 28446457 10.1136/bjsports-2017-097699

