# Supplementary material for: Temporal visual processing deficits in post concussion syndrome
Source: Sci Rep. 2025 Oct 15;15:36109. doi: 10.1038/s41598-025-24029-0 (PMC12528681; doi:10.1038/s41598-025-24029-0)
Supplement: Supplementary file 1 — Supplementary Material 1 [file 41598_2025_24029_MOESM1_ESM.pdf]

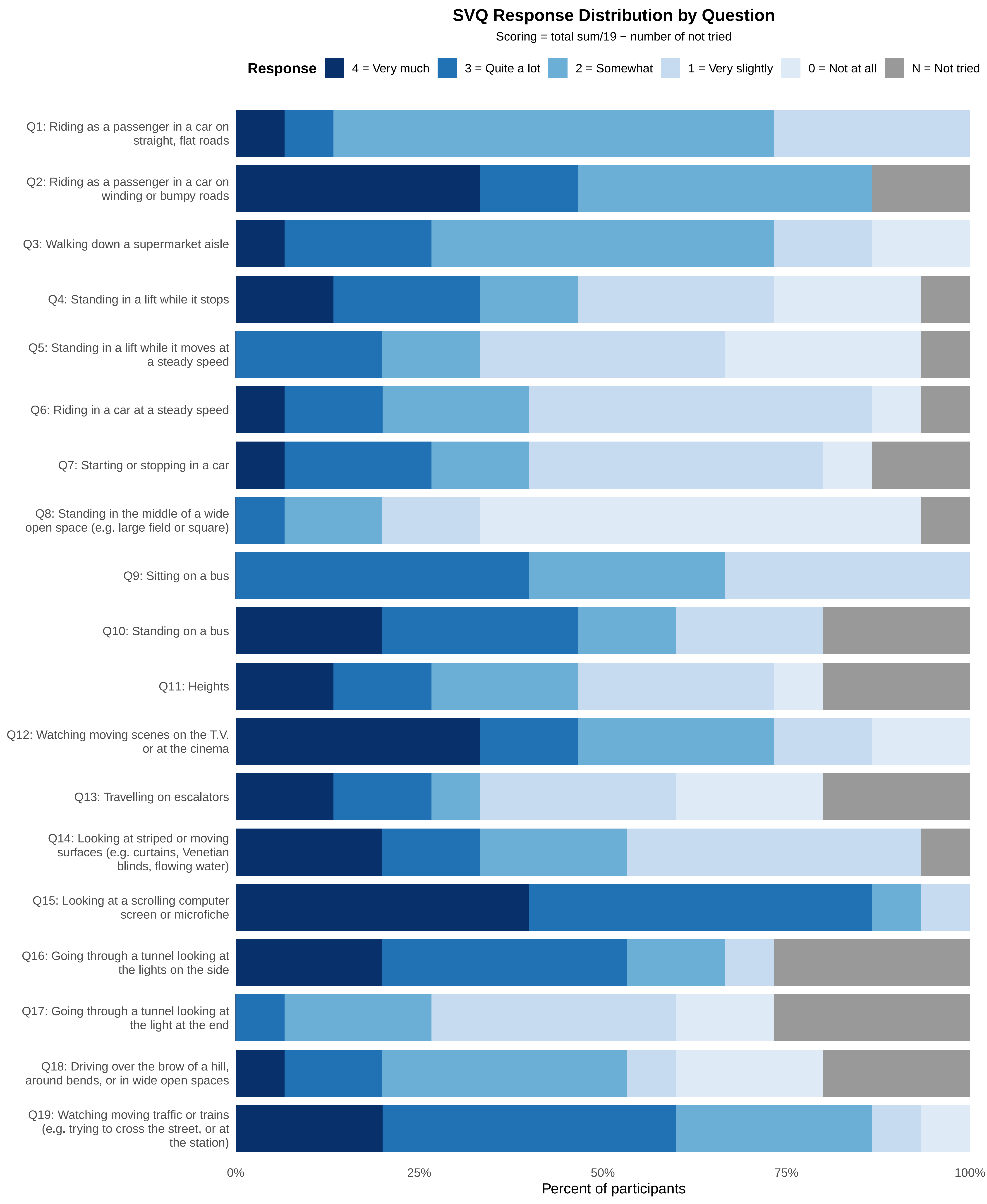

SVQ Response Distribution by Question
Scoring = total sum/19 − number of not tried
Response
4 = Very much
3 = Quite a lot
2 = Somewhat
1 = Very slightly
0 = Not at all
N = Not tried
Q1: Riding as a passenger in a car on straight, flat roads
Q2: Riding as a passenger in a car on winding or bumpy roads
Q3: Walking down a supermarket aisle
Q4: Standing in a lift while it stops
Q5: Standing in a lift while it moves at a steady speed
Q6: Riding in a car at a steady speed
Q7: Starting or stopping in a car
Q8: Standing in the middle of a wide open space (e.g. large field or square)
Q9: Sitting on a bus
Q10: Standing on a bus
Q11: Heights
Q12: Watching moving scenes on the T.V. or at the cinema
Q13: Travelling on escalators
Q14: Looking at striped or moving surfaces (e.g. curtains, Venetian blinds, flowing water)
Q15: Looking at a scrolling computer screen or microfiche
Q16: Going through a tunnel looking at the lights on the side
Q17: Going through a tunnel looking at the light at the end
Q18: Driving over the brow of a hill, around bends, or in wide open spaces
Q19: Watching moving traffic or trains (e.g. trying to cross the street, or at the station)
0%
25%
50%
75%
100%
Percent of participants
